# Core–shell microspheres for the ultrafast degradation of estrogen hormone at neutral pH†

**DOI:** 10.1039/c7ra11705a

**Published:** 2018-02-05

**Authors:** Katherine Villa, Jemish Parmar, Diana Vilela, Samuel Sánchez

**Affiliations:** Institute for Bioengineering of Catalonia (IBEC), The Barcelona Institute of Science and Technology Baldiri Reixac 10-12 08028 Barcelona Spain ssanchez@ibecbarcelona.eu +34 934 020 558; Max Planck Institute for Intelligent Systems Institution Heisenbergstraße 3 70569 Stuttgart Germany; Institució Catalana de Recerca i Estudis Avancats (ICREA) Pg. Lluís Companys 23 08010 Barcelona Spain

## Abstract

In the past few years there has been growing concern about human exposure to endocrine disrupting chemicals. This kind of pollutants can bioaccumulate in aquatic organisms and lead to serious health problems, especially affecting child development. Many efforts have been devoted to achieving the efficient removal of such refractory organics. In this regard, a novel catalyst based on the combination of α-FeOOH and MnO_2_@MnCO_3_ catalysts has been developed by up-scalable techniques from cheap precursors and tested in the photo-Fenton-like degradation of an endocrine disruptor. Almost total degradation of 17α-ethynylestradiol hormone was achieved after only 2 min of simulated solar irradiation at neutral pH. The outstanding performance of FeOOH@MnO_2_@MnCO_3_ microspheres was mainly attributed to a larger generation of hydroxyl radicals, which are the primary mediators of the total oxidation for this hormone. This work contributes to the development of more cost-effective systems for the rapid and efficient removal of persistent organic pollutants present in sewage plant effluents under direct solar light.

## Abstract 2

Ultrafast photo-Fenton degradation of 17α-ethynylestradiol under simulated solar irradiation.

## Graphical abstract

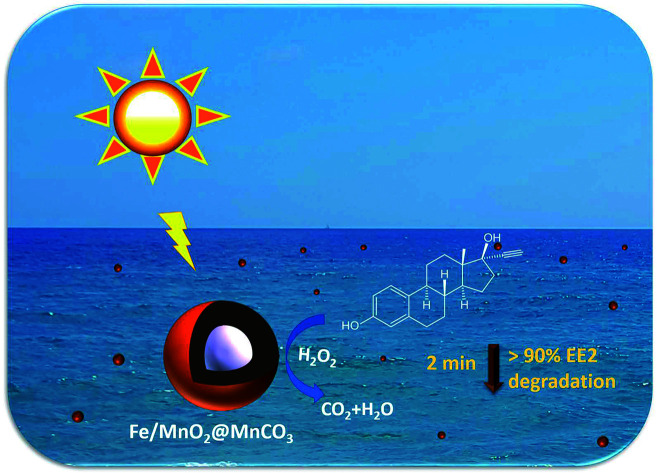

## Introduction

Industrial wastewater effluents from pharmaceuticals, agriculture, cosmetics, and personal care products usually contain a high amount of refractory organic pollutants.^1^ Among them, endocrine disrupting chemicals are considered a major concern for the aquatic environment and human beings, because of their ability to interact with the cell receptors of the body and mimic hormone-like activity. The presence of endocrine disruptors in water can lead to many health issues related to the reproductive system and immune disorders, even in very low concentrations.^2^ Considering that these toxic chemicals cannot be totally degraded by the classical bacteria-assisted water treatment methods and their tendency to accumulate in natural water and aquatic animals,^3^ it is essential to explore alternative catalytic approaches for their efficient removal in the water cleaning process.

One of the most powerful methods for this purpose is the Fenton reaction,^3–5^ which involves the generation of highly oxidizing species, known as hydroxyl radicals (HO˙), through the reaction between a dissolved iron salt (Fe^2+^) and hydrogen peroxide (H_2_O_2_), which can degrade all the organic molecules to CO_2_ and water. The performance of Fenton reaction can be improved by the so-called photo-Fenton degradation, where UV-visible light is used to generate extra reactive radicals from the photolysis of H_2_O_2_, and enhance the regeneration of the Fe^2+^ catalyst.^6,7^ Thus, the possibility of using solar light as energy source is an attractive way to transform it in a sustainable and more efficient technology.

Nevertheless, a real application of this advanced oxidation process is still hampered, mainly by the intrinsic limitations of the Fenton catalysts related to the requirement of an acidic pH. This entails extra costs associated with neutralization steps and further recovery of the formed iron sludges.

To address these problems, extensive recent research has focused on the development of heterogeneous iron-based catalysts that can activate H_2_O_2_ at neutral pH and facilitate recovery after the treatment process.^8–12^ However, the efficiency of these heterogeneous Fenton-like catalysts is usually found to be lower than its homogeneous counterpart. Thus, the design of heterogeneous catalysts that can effectively harvest solar light and decompose H_2_O_2_ into HO˙ without the need for a pH change is crucial for the practical application of solar Fenton-based wastewater treatments.

Among the catalysts reported for heterogeneous photo-Fenton-like reaction, amorphous iron oxide FeOOH (α-goethite) has shown an outstanding performance, even though its applicability has been limited by the slow Fe^3+^/Fe^2+^ cycle rates.^13–15^ Alternatively, other transition metal oxides, such as CeO_2_, CuO and MnO_2_, have also been reported as active materials for Fenton-like oxidation treatments.^16–18^ Among them, MnO_2_ (a non-toxic and cheap material) has not been extensively studied for the oxidation reaction of organic pollutants, despite being an efficient catalyst for the decomposition of H_2_O_2_.^19,20^

Here, we investigate for the first time the photo-Fenton-like degradation of 17α-ethynylestradiol hormone (EE2) using FeOOH@MnO_2_@MnCO_3_ core–shell catalyst. This material was synthesized by a facile surface oxidation of spherical MnCO_3_ crystal template to MnO_2_@MnCO_3_ and subsequent coating of α-FeOOH nanoparticles on it. Since MnO_2_ facilitates the fast decomposition of H_2_O_2_ and iron cycling, the combination of α-FeOOH and MnO_2_ results in a great enhancement of the overall catalytic process. Additionally, the performance of this material can be improved by using mainly visible light, which is the major component of the solar radiation. The outcome of this research would contribute to the feasibility of the photo-Fenton-like technology by using Fe/Mn microspheres-based system for the treatment of contaminated water with industrial pollutants.

## Experimental

### Catalyst preparation

**Fig. 1:**
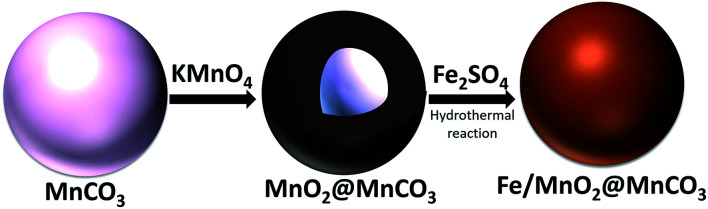
Schematic illustration of the synthesis procedure of Fe/MnO_2_@MnCO_3_ catalyst.

MnCO_3_ microparticles were synthesized by mixing 70 mL of 0.014 M MnSO_4_ solution (ACS reagent ≥99.0%, Sigma-Aldrich) with 0.79 g of NH_4_CO_3_ (BioUltra ≥99.5%, Sigma-Aldrich) at room temperature for 15 h.^21^ A schematic illustration of the synthesis procedure of α-FeOOH@MnO_2_@MnCO_3_ microspheres is shown in Fig. 1. A core–shell structure of MnO_2_@MnCO_3_ was prepared by mixing 0.09 g of the as-synthesized MnCO_3_ with 6.0 mL of aqueous solution of 0.032 M KMnO_4_ (ACS reagent ≥99.0%, Sigma-Aldrich) for 4 h. The sample was collected by centrifugation and washed with Milli-Q water. Afterwards, the MnO_2_@MnCO_3_ sample was dispersed in 30 mL of glycerol–water mixture by sonication for 20 min. FeOOH was grown on the MnO_2_@MnCO_3_ microparticles by adding 0.166 g of FeSO_4_·7H_2_O (ACS reagent ≥99.0%, Sigma-Aldrich) to that mixture and stirring for 10 min. Afterwards, the black resulting suspension was transferred into a 50 mL Teflon-lined stainless-steel autoclave and heated at 120 °C for 24 h. The dark orange precipitate was collected by centrifugation and washed sequentially with water and ethanol, and dried at 60 °C for 6 h. A control synthesis experiment was carried out by adding the iron precursor (in the absence of MnO_2_@MnCO_3_ microparticles) to confirm that the iron oxide phase grown by the hydrothermal reaction consists of α-FeOOH.

For the sake of simplicity, the FeOOH@MnO_2_@MnCO_3_ material has been labeled as Fe/MnO_2_@MnCO_3_.

### Catalyst characterization

The crystal structure of the as-synthesized samples was determined by X-ray powder diffraction (XRD) using a Bruker D8 Advance diffractometer equipped with a Cu Kα radiation (1.5417 Å) source, a LYNXEYE super speed detector and a Ni filter. X-ray diffraction data was collected in the 2*θ* range between 20° and 80° using a scan rate of 0.05° by 2 s. The light absorption properties were measured by using a UV-Vis diffuse reflectance spectrophotometer (Perkin Elmer Lambda 950 UV-Vis) with a wavelength range of 250–800 nm. A Zeiss Auriga microscope equipped with energy-dispersive X-ray (EDX) detector was used to perform scanning electron microscopy (SEM) and EDX analysis.

X-ray photoelectron spectroscopy (XPS) from SPECS system (Germany) was used to identify Mn and Fe oxidation states on the samples. The instrument was equipped with XR50 duel anode source (Al operated at 150 W) and a Phoibos MCD-9 detector. All measurements were done under the vacuum (pressure 5 × 10^−9^ mbar) and the hemispherical analyzer was set at the pass energy 25 eV while the high resolution spectra step size was set at 0.1 eV. Casa XPS program (Casa Software Ltd., UK) was used for the data analysis.

### Photo-Fenton set-up

The degradation of an aqueous solution of EE2 was carried out in a 10 mL capacity cylindrical glass vessel. The catalyst slurries were magnetically stirred during the reaction. For the photo-Fenton-like experiments, a 300 W high pressure UV-visible lamp (Ultravitalux Osram, 280–780 nm) was used to simulate solar irradiation. In a typical experiment, 6 mL of the 0.5 ppm of EE2 solution containing 0.0015 g of catalyst was placed in the reactor. Since the main aim of this study was to evaluate the catalytic activity at near-neutral pH (6.7), no acidic or basic pH adjustment was performed.

Prior to illumination, the suspension was sonicated in the dark for 30 min to reach adsorption–desorption equilibrium. After that, the lamp was turned on and H_2_O_2_ was added to initiate the reaction. Liquid samples were periodically taken out, immediately centrifuged, and then analyzed by high performance liquid chromatography (Acquity) coupled to a triple quadrupole mass spectrometer LC/MS/MS (API 3000). The mobile phase consisted of 50 : 50 v/v ultrapure water : acetonitrile mixture and the injection volume of the sample was 10 μL. In the case of the MB degradation, the concentration was monitored by measuring its absorbance at 664 nm (*λ*_max_) during the reaction by using a UV-Vis spectrophotometer (Specord 50 Plus).

A blank test without catalyst (only H_2_O_2_ + light irradiation) was also performed to evaluate the contribution of the photolysis of H_2_O_2_, which involves the generation of hydroxyl radicals, on the oxidation of our target molecule (EE2). Additionally, a test with catalyst under light irradiation and no H_2_O_2_ was carried out to estimate the photocatalytic influence on the resulting performance.

The hydroxyl radical generation during the photo-Fenton-like reaction was identified by following a fluorescence method, using 5 × 10^−4^ M terephthalic acid (TA) as a chemical trap of HO˙. The excitation wavelength was 320 nm and the fluorescence emission spectra were acquired from 350–600 nm using a multimode microplate reader (Infinite M200 PRO). The experimental conditions were the same as those described above for the irradiation tests and H_2_O_2_ concentration was 0.1 M.

## Results and discussion

**Fig. 2:**
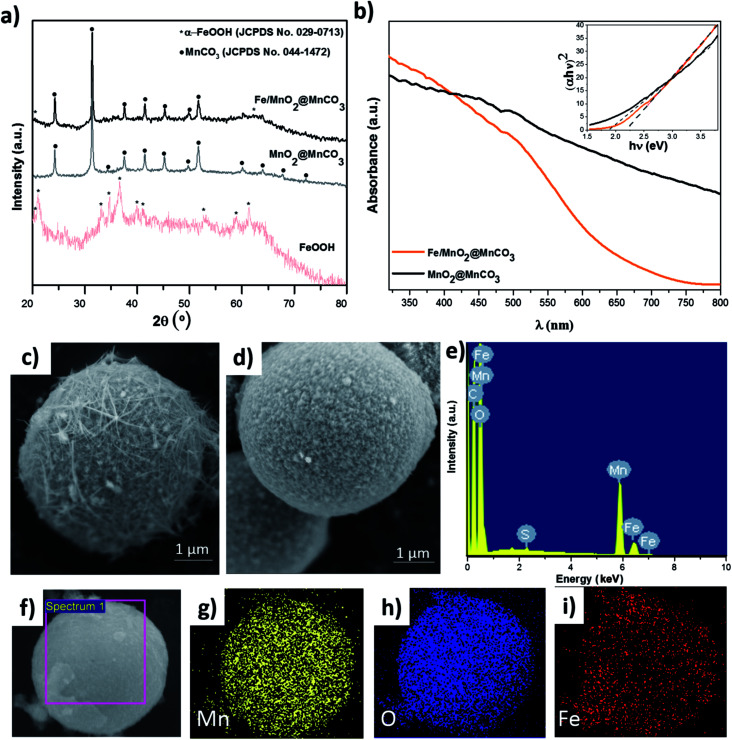
Characterization of materials. (a) X-ray powder diffraction patterns of α-FeOOH, MnO_2_@MnCO_3_ and Fe/MnO_2_@MnCO_3_, (b) UV-Vis diffuse reflectance spectra (DRS) of MnO_2_@MnCO_3_ and Fe/MnO_2_@MnCO_3_. In the inset, the Tauc plot of (absorbance energy)^2^ against energy obtained from DRS UV-Vis spectra, (c) SEM image of MnO_2_@MnCO_3_, (d) SEM image of Fe/MnO_2_@MnCO_3_, (e) EDX spectra of Fe/MnO_2_@MnCO_3_, (f–i) EDX mapping of Fe/MnO_2_@MnCO_3_.

To study the composition and structure of the catalysts, different characterization techniques such as XRD, UV-Vis and SEM/EDX were used (Fig. 2). As observed in XRD patterns displayed in Fig. 2a, MnO_2_@MnCO_3_ only presents diffraction peaks corresponding to the crystalline structure of MnCO_3_ (JCPDS no. 044-1472). Since the intensity of the core peaks was too high, the external shell of MnO_2_ was not appreciated, which is in agreement with previously reported works.^20,21^ The XRD patterns of the iron oxide obtained by the hydrothermal reaction indicate the formation of α-goethite FeOOH phase (JCPDS no. 029-0713). In the case of Fe/MnO_2_@MnCO_3_ microparticles, the diffraction peaks are very similar to those of bare MnO_2_@MnCO_3_, being also difficult to detect the peaks associated with MnO_2_. However, an additional peak at around 20.1° and a shoulder at approximately 62.1° can be attributed to the α-FeOOH phase from the surface of Fe/MnO_2_@MnCO_3_ microspheres.

A slight shift in the optical absorption of bare MnO_2_@MnCO_3_ catalyst was observed in the UV-Vis diffuse reflectance spectra after being loaded with α-FeOOH (Fig. 2b). The band gap energy (*E*_g_) of the materials was calculated from the absorption data by using the Tauc plots (see inset, Fig. 2b), where (absorbance energy)^2^ is plotted against energy.^22^ The estimated values of 1.9 and 2.2 eV for MnO_2_@MnCO_3_ and Fe/MnO_2_@MnCO_3_ catalysts, respectively, confirm that the optical absorption properties of these Mn-based materials lie in the visible region. Considering a real application under direct solar light, this is advantageous in comparison with other widely studied materials such as TiO_2_, which can only be activated by ultraviolet light.^23,24^

SEM images are shown in Fig. 2c and d. MnO_2_@MnCO_3_ microparticles consist of a spherical shape with an approximate diameter of 4.5 μm and a fibrous surface. It is worth noting that due to their large size compared to nanometric particles, these microparticles can be easily recovered from the solution after each catalytic reaction. In the case of the Fe/MnO_2_@MnCO_3_, the spherical shape, size, and roughness were preserved (Fig. 2d). However, due to the formation of the external α-FeOOH coating on the surface, it presents a higher homogeneity than MnO_2_@MnCO_3_. The EDX spectrum confirmed the presence of Fe in the Fe/MnO_2_@MnCO_3_ catalyst structure (Fig. 2e), in agreement with XRD characterization. Furthermore, EDX-mapping showed the uniform distribution of Fe on the catalyst surface (Fig. 2f–i).

**Fig. 3:**
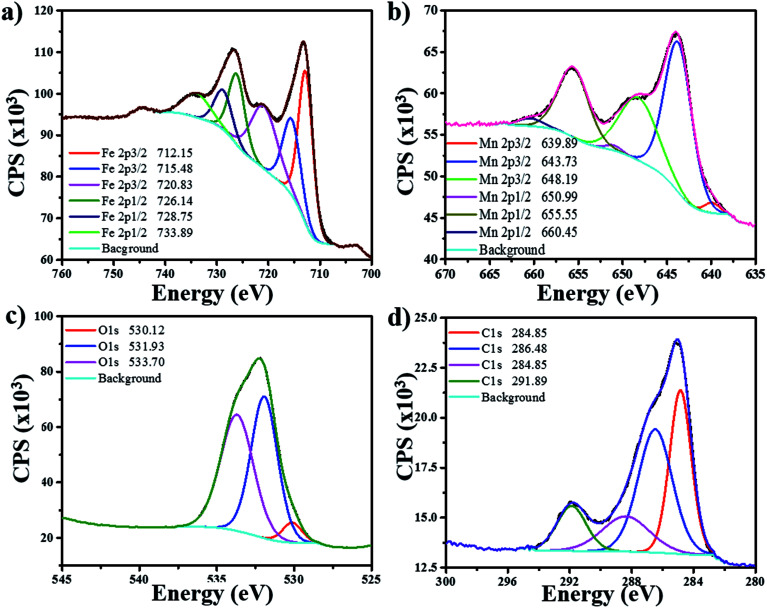
XPS spectra of Fe/MnO_2_@MnCO_3_ catalyst for its different elements: (a) iron (Fe), (b) manganese (Mn), (c) oxygen (O), (d) carbon (C).

The chemical states of Fe and Mn in Fe/MnO_2_@MnCO_3_ sample were investigated by XPS. Fig. 3a shows the XPS spectrum of Fe 2p. The complex multiplet-split of Fe 2p regions is typical of Fe^3+^ compounds.^25^ The two main peaks at 712.75 and 726.14 eV can be assigned to Fe 2p_3/2_ and Fe 2p_1/2_ signals from FeOOH. Additionally, the satellite peak of Fe 2p_3/2_ at 720.83 eV would confirm the presence of Fe^3+^ ions in the composite.^26,27^ In the case of Mn 2p (Fig. 3b), two main peaks at 643.73 and 655.55 eV are distinguished, corresponding to Mn 2p_3/2_ and Mn 2p_1/2_, respectively. These two spin–orbit components have a separation of approximately 12 eV, evidencing the presence of Mn^4+^ from the MnO_2_ material.^26^ An additional Mn 2p_3/2_ satellite peak at 648.19 eV can be attributed to the presence of MnO (MnOOH oxide),^25^ resulting from the hydrothermal treatment in the presence of FeOOH precursors. This fact evidences a mixture of valence states (Mn^3+^ and Mn^4+^) in the Fe/MnO_2_@MnCO_3_ catalyst. The XPS O 1s region shows three contributions at 530.12, 531.93 and 533.70 eV (Fig. 3c). The first one can be attributed to the oxygen atoms in the lattice of metal–oxygen structure. The second one at 531.93 eV corresponds to the metal-hydroxyl bond and the latter at 533.70 eV is assigned to the water adsorbed on the catalyst surface.^28–30^ Finally, the spectrum from C 1s display a high signal observed at 291.8 eV (Fig. 3d) may be assigned to the carbonate groups (CO_3_^2−^) present in the sample, due to the composite core (MnCO_3_).

A preliminary screening of the bare catalysts and the composite was carried out at neutral pH using methylene blue (a common dye waste). As presented in Fig. S1,† the performance of FeOOH catalyst is almost negligible, whereas Fe/MnO_2_@MnCO_3_ composite shows a remarkable Fenton-like activity compared to MnO_2_@MnCO_3_ material. Based on this, FeOOH photocatalyst was not considered for further degradation experiments.

The photo-Fenton-like degradation of EE2, as an example of a persistent endocrine disrupting pollutant, was studied in an aqueous suspension containing the as-synthesized MnO_2_@MnCO_3_ and Fe/MnO_2_@MnCO_3_ catalysts in the presence of H_2_O_2_, under UV-visible light irradiation. Considering that endocrine disrupting chemicals are usually found at low concentrations in sewage plant effluents, and taking into account that even at those low levels they are toxic,^31,32^ we selected a 0.5 ppm EE2 concentration for the degradation tests.

**Fig. 4:**
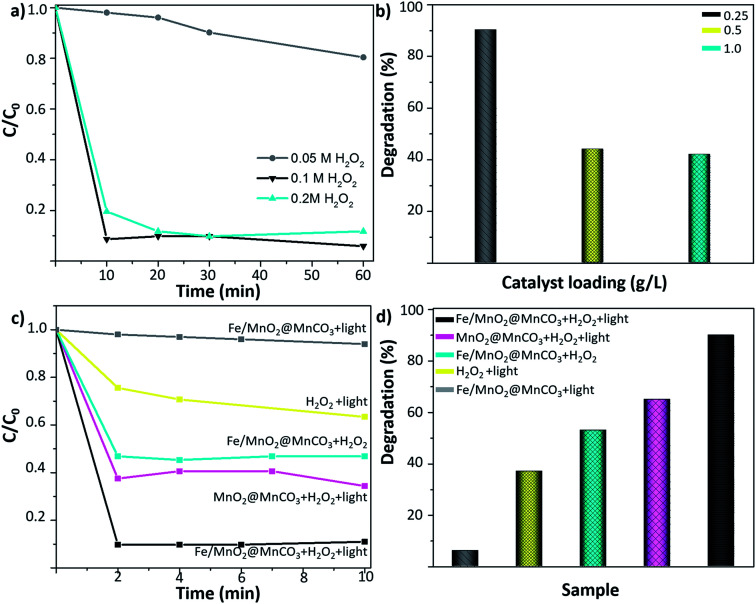
Photo-Fenton-like degradation of 17α-ethynylestradiol. (a) Effect of H_2_O_2_ concentration (0.25 g L^−1^ catalyst loading) and (b) effect of catalyst loading (0.1 M H_2_O_2_), (c) *C*/*C*_0_ kinetics and (d) photo-Fenton-like degradation of 17α-ethynylestradiol after 10 min of reaction under different conditions: Fe/MnO_2_@MnCO_3_ + light, H_2_O_2_ + light, Fe/MnO_2_@MnCO_3_ + H_2_O_2_ (in the dark), MnO_2_@MnCO_3_ + H_2_O_2_ + light, and Fe/MnO_2_@MnCO_3_ + H_2_O_2_ + light.

Initially, the concentration of H_2_O_2_ was optimized. As shown in Fig. 4a, the degradation of 0.5 ppm EE2 enhanced from 10 to 90% by increasing the H_2_O_2_ concentration from 0.05 M to 0.1 M. However, a further increase to 0.2 M did not lead to an additional improvement of the degradation rates, evidencing that a plateau was reached. Therefore, 0.1 M H_2_O_2_ was selected as the optimal minimum concentration for further experiments. Afterwards, to select the optimal amount of catalyst, different catalyst loadings from 0.25 to 1 g L^−1^ were tested (Fig. 4b). The highest degradation value was obtained by using the lowest amount of catalyst (0.25 g L^−1^). This fact can be attributed to the severe light scattering effect provoked by larger catalyst concentrations, which reduces the number of available photons able to reach each catalyst particle,^33,34^ resulting in a decrease of the radical species generation and a subsequent decrease in the degradation efficiency of the EE2 hormone.

Fig. 4c and d show the degradation kinetics of 0.5 ppm EE2 compound after 10 min of UV-visible irradiation. Control experiments consisting of the irradiation of Fe/MnO_2_@MnCO_3_ (without H_2_O_2_) and 0.1 M H_2_O_2_ (without catalyst) were carried out under the optimized reaction conditions. It was found that the photocatalytic activity of Fe/MnO_2_@MnCO_3_ without H_2_O_2_ only contributed to around 6% of the degradation after 10 min. Furthermore, it is well-known that the irradiation of H_2_O_2_ with UV-visible irradiation also leads to hydroxyl radicals generation by photolysis.^35^ Nonetheless, under those conditions the degradation yield was also low and slow. It is worth highlighting that the high pressure lamp used in this work (maximum intensity at 365 nm) cannot lead to the photodegradation of the synthetic hormone.^36^ When the photo-Fenton tests were performed in the presence of MnO_2_@MnCO_3_ and Fe/MnO_2_@MnCO_3_ catalysts and 0.1 M H_2_O_2_, the degradation rates were noticeably enhanced after only two minutes of light irradiation. This result shows that the presence of the catalyst is required to improve the decomposition of H_2_O_2_ and accelerate the degradation rate.

The MnO_2_@MnCO_3_ catalyst showed a 62% EE2 degradation within 2 min of the photo-Fenton-like reaction, and that maximum degradation yield remained constant over time, suggesting that the catalyst is rapidly consuming all the H_2_O_2_ to generate O_2_. Moreover, unlike the Fe-based catalysts, MnO_2_ is more prone to generate superoxide and hydroperoxy radicals,^37^ which have a lower oxidation potential than the hydroxyl radicals.^38^

As shown in Fig. 4d, the highest degradation yield was achieved with the Fe/MnO_2_@MnCO_3_ microspheres. This improvement in the photo-Fenton performance might be explained by multiple factors, such as the synergistic effect arising from the activity of manganese and iron metal oxides in the photo-Fenton-like reaction. In addition, it has been reported that Fe-based catalysts are not efficient in activating H_2_O_2_ at high pH values,^39–42^ while MnO_2_ is able to efficiently decompose H_2_O_2_ molecules even at neutral pH.^37^ Due to this combination, the resulting Fe/MnO_2_@MnCO_3_ composite achieved more than 90% degradation of the hormone at near-neutral pH (6.7) in just 2 min of light irradiation. Given the limit of quantification of the equipment, degradations above 90% could not be measured reliably (see ESI, Fig. S2†).

Furthermore, this approach was compared to previous works about Fenton-like and photo-Fenton-like degradation of EE2. For instance, Park *et al.* reported a 90% degradation of 0.02 ppb of this hormone after 8 h of Fenton-like reaction with Ag nanoparticles at pH 4,^43^ while in our study, a 53% degradation (0.5 ppm EE2 concentration) value was obtained with our material after 10 min of Fenton-like reaction at near-neutral pH. Even in a homogeneous photo-Fenton reaction, which is usually considered more efficient than the heterogeneous one, 80% degradation was reported after 160 min of UV-visible irradiation at pH 3.^44^ Thus, Fe/MnO_2_@MnCO_3_ catalyst is a promising material for the ultrafast degradation of endocrine disruptor chemicals in contaminated water without the need of an acidic pH.

**Fig. 5:**
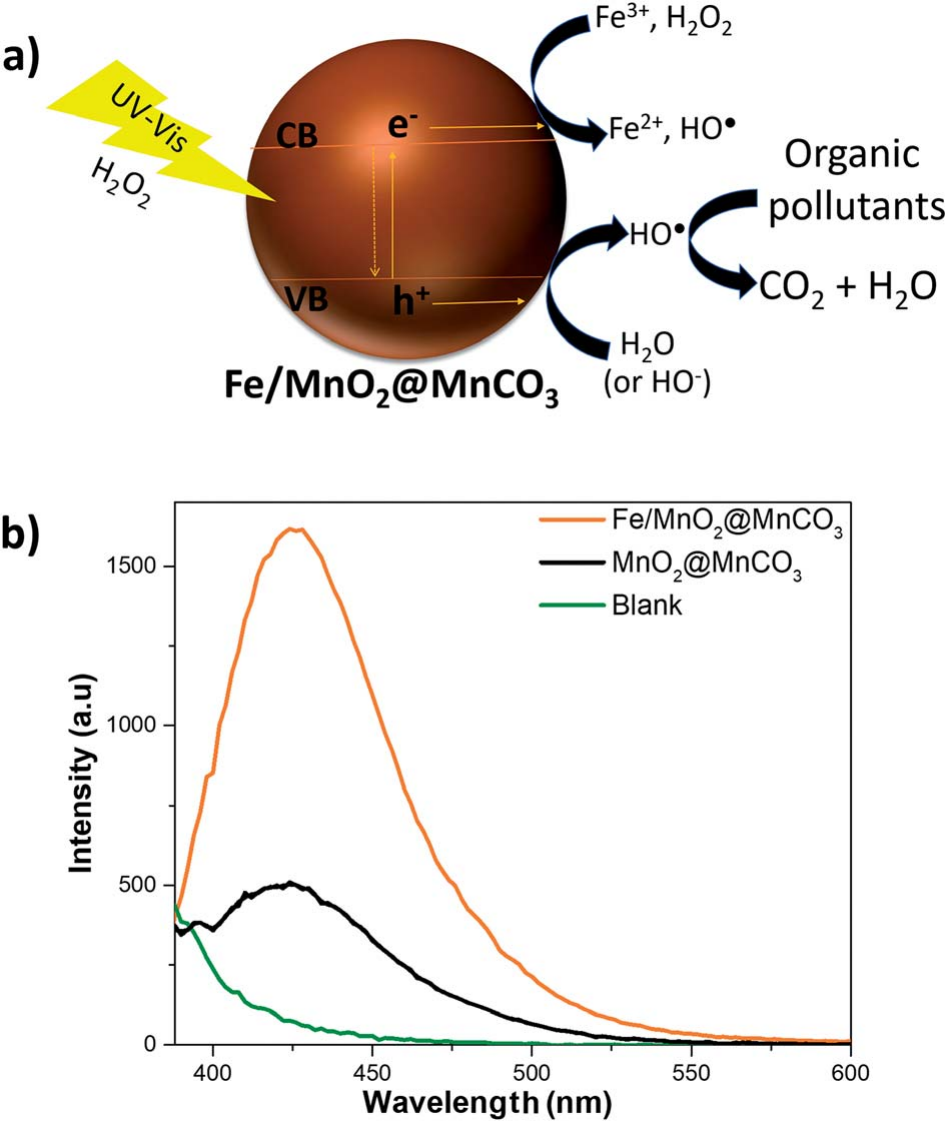
Insight into the photo-Fenton-like degradation mechanism of EE2. (a) Scheme of the photo-Fenton-like mechanism of Fe/MnO_2_@MnCO_3_ and (b) fluorescence spectra of HTA in the presence of MnO_2_@MnCO_3_ and Fe/MnO_2_@MnCO_3_ after 10 min of irradiation.

Although the mechanism of the photo-Fenton-like reaction is still not fully understood, it is widely accepted that it involves mainly the generation of hydroxyl radicals (highly reactive species), which are the key players in the oxidation of organic compounds to CO_2_ and water.^45^ As observed in Fig. 5a, after activation of the Fe/MnO_2_@MnCO_3_ catalyst with light energy higher than ∼2.2 eV, electron (e^−^) and hole (h^+^) pairs are generated. Then, these photo-generated holes can react with water or hydroxide ions adsorbed on the catalyst surface to produce hydroxyl radicals. In addition, the electrons can contribute to enhancing the reduction of Fe^3+^ to Fe^2+^ ions (limiting step), and react with H_2_O_2_ to form additional HO˙ radicals. Moreover, Mn^4+^ and Mn^3+^ ions help to the recycling of Fe^2+^ to Fe^3+^ species by electron transfer. Finally, the reduced Mn^2+^ ions are oxidized again by the H_2_O_2_ adsorbed on the catalyst surface.^46^

Ohko *et al.*^47^ studied the photocatalytic degradation mechanism of 17β-estradiol (E2) over TiO_2_ photocatalyst. 10ε-17β-Dihydroxy-1,4-estradien-3-one and testosterone-like compounds were detected as intermediary products, which were further oxidized to CO_2_. The whole oxidation pathway was mainly mediated by hydroxyl radicals, starting from the oxidation of the phenol group. Since the molecular configuration of EE2 (steroid structure with a phenol group) is similar to E2 hormone, it can be expected that its degradation mechanism follows the same pathway.

Consequently, to evaluate the radical species produced during the photo-Fenton-like reaction, we examined the formation of hydroxyl radicals using TA to specifically trap these reactive species, and produce 2-hydroxyterephthalic acid (HTA), which exhibits fluorescence emission at 425 nm.^48,49^ The fluorescence spectra corresponding to the formation of HTA in the presence of the catalysts are shown in Fig. 5b. The signal intensity associated with the formation of HO˙ species was almost 3 times higher in the presence of Fe/MnO_2_@MnCO_3_ in comparison with bare MnO_2_@MnCO_3_. This result clearly confirms that the α-FeOOH coating promotes a higher production of hydroxyl radicals on MnO_2_@MnCO_3_ microparticles, resulting in an enhancement of the degradation of EE2 hormone.

**Fig. 6:**
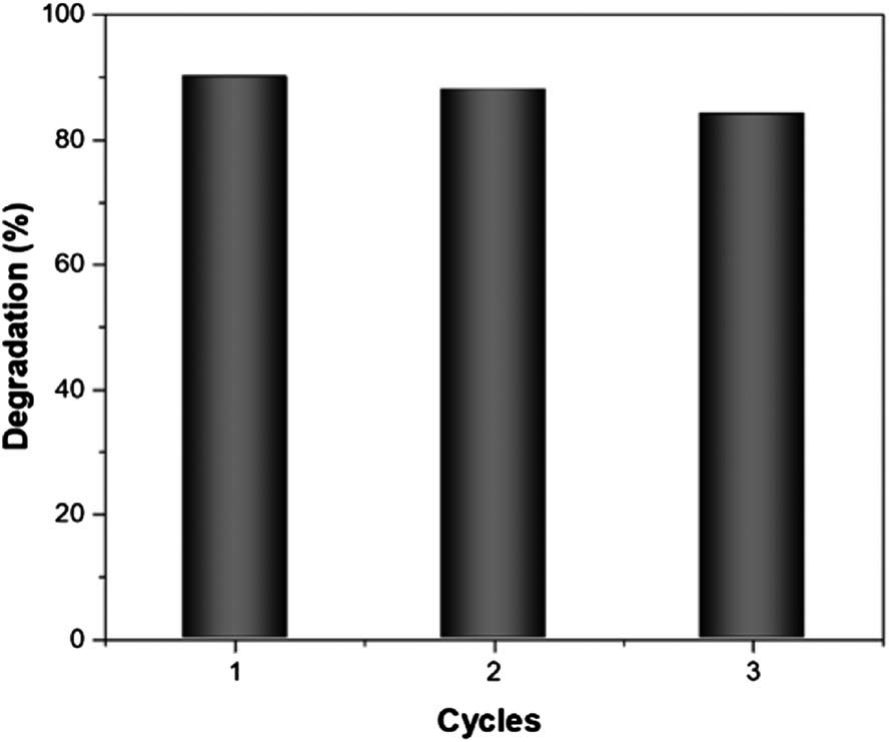
Reuse of Fe/MnO_2_@MnCO_3_ catalyst in the photo-Fenton-like degradation of EE2 after 10 min of irradiation. Reaction conditions: EE2 (0.5 ppm), H_2_O_2_ concentration 0.1 M. Catalyst loading 0.25 g L^−1^.

Finally, the reusability of Fe/MnO_2_@MnCO_3_ catalyst was studied in the photo-Fenton-like degradation of EE2 after 10 min of simulated solar irradiation under 3 consecutive cycles. After each test, the sample was recovered by centrifugation and reused with a fresh solution of the hormone. As can be seen in Fig. 6, the catalytic activity decreased by only 6% between the first and third cycle. This result might be due to a blockage of the active sites by the intermediaries formed during the degradation process that remain adsorbed on the catalyst surface.^50,51^ Based on these results, it seems fair to suggest that this material exhibits a good reusability, which along with its low-cost and up-scalable synthesis, results in a cost-effective catalyst for the efficient degradation of organic pollutants from industry wastewaters.

## Conclusions

To sum up, we have developed a novel cost-effective and reusable composite based on iron/manganese microspheres, which has demonstrated to be an efficient heterogeneous catalyst for the fast photo-Fenton-like degradation of 17α-ethynylestradiol hormone, under simulated solar irradiation. Considering that its synthesis process involves precipitation reaction at room temperature and hydrothermal steps from cheap precursors, this material can be easily obtained at a large scale. The synergy between FeOOH and MnO_2_ significantly improves the resulting performance due to a fast activation of H_2_O_2_ and a larger generation of hydroxyl radicals, thus achieving more than 90% degradation of the hormone within 2 minutes of reaction at near-neutral pH. Moreover, the relatively large size of the resulting composite facilitates the recovery step after each catalytic reaction. Besides this, since the light absorption of Fe/MnO_2_@MnCO_3_ catalyst lies in the visible region, this material can be successfully used for the practical application of solar driven Fenton-like systems for the treatment of industrial organic pollutants, without the need of an acidic pH.

## Conflicts of interest

There are no conflicts to declare.

## Supplementary Material

RA-008-C7RA11705A-s001
