# Supplementary material for: Core–shell microspheres for the ultrafast degradation of estrogen hormone at neutral pH
Source: RSC Adv. 2018 Feb 5;8(11):5840–7. doi: 10.1039/c7ra11705a (PMC9078169; doi:10.1039/c7ra11705a)
Supplement: RA-008-C7RA11705A-s001 [file RA-008-C7RA11705A-s001.pdf]

Electronic Supplementary Material (ESI) for RSC Advances.
This journal is © The Royal Society of Chemistry 2018

Supplementary Information

Core-shell microspheres for the ultrafast degradation of estrogen hormone at neutral pH

Katherine Villa [a], Jemish Parmar [a, b], Diana Vilela [a, b], Samuel Sánchez [a, b, c†]*

[†] Institute for Bioengineering of Catalonia (IBEC), The Barcelona Institute of Science and Technology, Baldiri Reixac 10-12, 08028 Barcelona Spain

[‡] Max Planck Institute for Intelligent Systems Institution, Heisenbergstraße 3, 70569 Stuttgart, Germany.

[§] Institució Catalana de Recerca i Estudis Avancats (ICREA), Pg. Lluís Companys 23, 08010, Barcelona, Spain

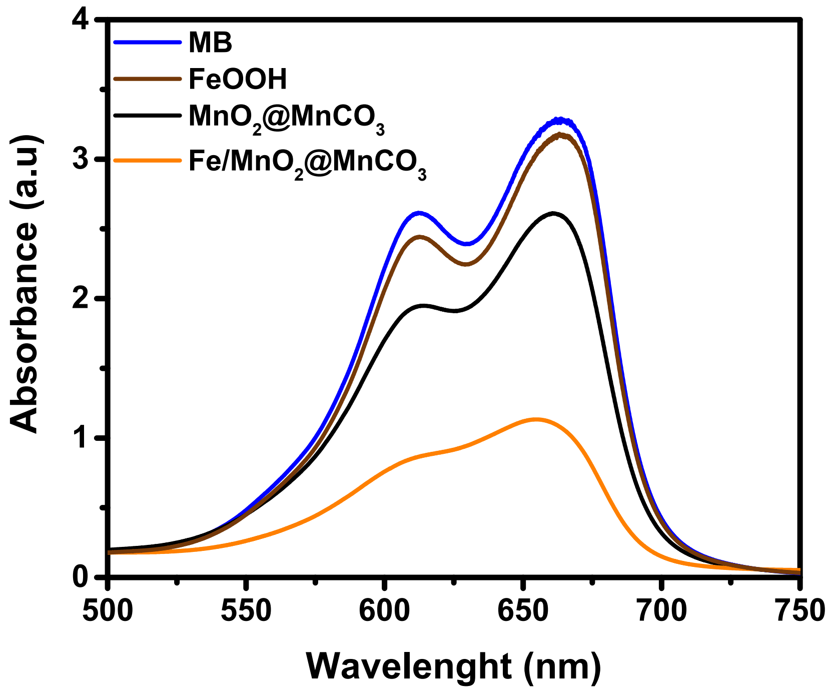

Figure S1. a) Degradation of MB (20 µg/ml) after 2 h of reaction. Reaction conditions: MB (20 µg/L), H_2O_2 concentration 0.1 M. Catalyst loading 0.25 g/L.

Measurement of 17α-ethynylestradiol by HPLC coupled to Mass spectrometry

A calibration curve for 17α-ethynylestradiol (EE2) hormone was carried out by HPLC/Mass spectrometry for the concentration range from 0-0.5 ppm (Figure S1). Based

on the data obtained, we calculated the limit of detection (LOD) and quantification (LOQ) of the equipment for EE2 as being 0.014 ppm and 0.045 ppm, respectively. Since the initial concentration of EE2 used in the degradation experiments was 0.5 ppm, further degradation values larger than 90% could not be determined by the equipment.

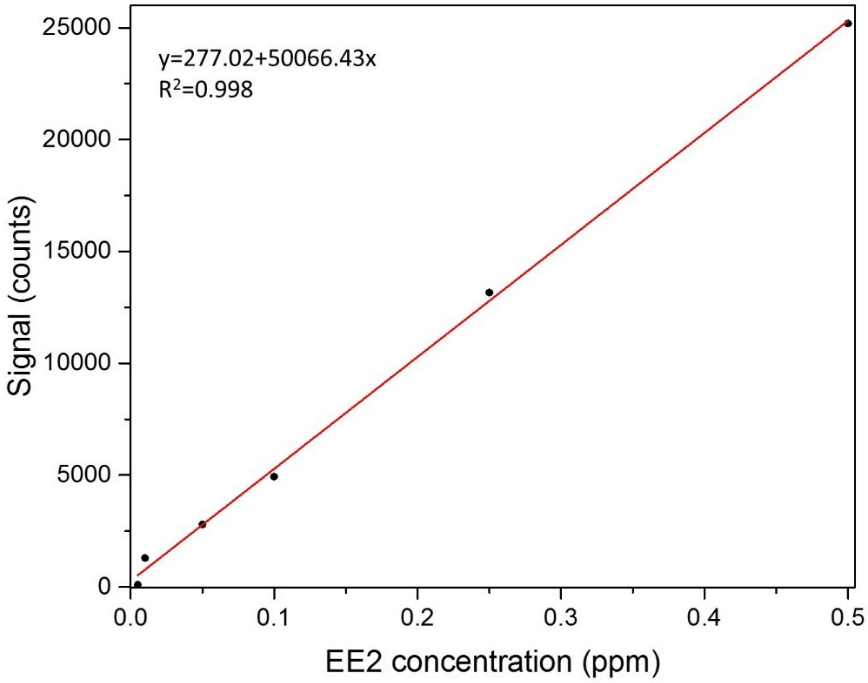

Figure S2. Calibration curve of EE2 hormone.
